# A Systematic Review of IoT Solutions for Smart Farming

**DOI:** 10.3390/s20154231

**Published:** 2020-07-29

**Authors:** Emerson Navarro, Nuno Costa, António Pereira

**Affiliations:** 1 School of Technology and Management, Computer Science and Communication Research Centre, Polytechnic Institute of Leiria, Campus 2, Morro do Lena—Alto do Vieiro, Apartado 4163, 2411-901 Leiria, Portugal; 2180233@my.ipleiria.pt (E.N.); nuno.costa@ipleiria.pt (N.C.); 2 INOV INESC Inovação, Institute of New Technologies, Leiria Office, Campus 2, Morro do Lena—Alto do Vieiro, Apartado 4163, 2411-901 Leiria, Portugal

**Keywords:** smart farming, IoT technologies, big data, artificial intelligence, cloud computing

## Abstract

The world population growth is increasing the demand for food production. Furthermore, the reduction of the workforce in rural areas and the increase in production costs are challenges for food production nowadays. Smart farming is a farm management concept that may use Internet of Things (IoT) to overcome the current challenges of food production. This work uses the preferred reporting items for systematic reviews (PRISMA) methodology to systematically review the existing literature on smart farming with IoT. The review aims to identify the main devices, platforms, network protocols, processing data technologies and the applicability of smart farming with IoT to agriculture. The review shows an evolution in the way data is processed in recent years. Traditional approaches mostly used data in a reactive manner. In more recent approaches, however, new technological developments allowed the use of data to prevent crop problems and to improve the accuracy of crop diagnosis.

## 1. Introduction

The challenge of food production in the 21st century is an increasingly relevant theme as population growth increases year after year. It is estimated that by 2050 the world will have between 9.4 and 10.1 billion people who depend on the world’s biodiversity to live, increasing the demand for dedicated food production areas—specifically for planting and livestock [1]. Environmental changes caused by human beings could potentially cause conditions in which the development of new crops is not possible. Likewise, the growing urbanization decreases labor in areas typically involved in food production, increases costs and reduces the productive capacity of the sector [2]. In view of this, smart farming is a new farm management concept that employs techniques and technologies at various levels and scales of agricultural production, enabling to overcome the challenges in food production demands and reduction in the workforce [3,4]. For example smart farming may use different types of sensors to collect data (e.g., temperature, humidity, light, pressure, presence, etc.), communication networks to send and receive data which is then managed and analyzed by management information systems and data analysis solutions [5]. This system of interconnected devices is commonly referred to as internet of things (IoT) [6]. The use of the data provided by smart farming helps boosting productivity and minimizing waste by allowing necessary actions to be carried out at the right time, quantity and place [7].

Moreover, recent technological developments in areas relevant to IoT facilitates an easier adoption and use of smart farming with IoT [8]. Such technological developments include, for example, network communications, reduction of hardware size, optimization of power consumption and devices cheapening. Furthermore, the World’s largest agricultural producers are promoting the usage of IoT in smart farming by creating incentive programs and public policies to fund research and training [9,10].

Several reviews have been published on IoT solutions for smart agriculture in recent years which denotes that this research field is being constantly receiving new contributions and constant improvement. Existing reviews usually focus on topics like network technologies, embedded system platforms, unmanned aerial vehicles (UAV) devices, network protocols and topologies and enabling cloud platforms. For instance [11] focuses on arable farming from year 2008 to 2018 and surveys communication technologies and protocols, the generation and analysis of data, IoT architectures and applications and highlights the challenges and future directions related with the application of IoT technologies on arable farming. Review [12] presents technologies used for communication and data collection within IoT solutions for smart farming as well as several cloud-based IoT platforms used for IoT solutions for smart farming. Additionally, authors present several use cases for the identified applications of IoT for smart farming. Review [13] presents a systematic review of papers published between 2006 and 2016 and classifies these papers in application domains, such as monitoring, controlling, logistic and prediction. Within these domains, authors also identified the data visualization strategies and the technologies used for communication and edge computing. Review [14] presents a review of papers published between 2010 and 2016. The authors rely on an IoT architecture with three layers (perception, network, application) to analyze the reviewed papers in terms of perception devices, network technologies and applications. With this, they identify embedded platforms and communication technologies used in IoT solutions as well as the application of such IoT solutions. Finally, [15] reviewed papers published between 2010 and 2015 and presents a state-of-the-art of IoT solutions for smart farming and smart agriculture. Authors relied on an IoT architecture with three layers (perception, network and application) to analyze the application of sensor and actuator devices and communication technologies within several farming domains, such as agriculture, food consumption, livestock farming, among others.

This study aims to identify how IoT is used with smart farming by (i) presenting a systematic review of the state of the art of the IoT adoption in smart agriculture and (ii) identifying the most commonly used hardware, platforms, network protocols and technologies and their applicability to the proposed solutions. To give a more up-to-date view to the reader about the smart agriculture research field, this review article surveys literature until year 2020 and evaluates the acceptance of (new) IT technologies, such as big data, computer vision, artificial intelligence, blockchain and fuzzy logic in the smart agriculture field. This review complements the analysis already carried out by the academy by performing a comprehensive review of the state-of-the-art of IoT in smart farming. To do so, we rely on an IoT architecture with 4 layers (perception, network, processing and application) to identify the technologies that enable IoT in smart farming. From this approach, the present work identifies an increasing use of modern techniques and technologies for processing the collected data in recent years, such as big data, machine learning, computer vision and blockchain. Moreover, this review contributes to the existent literature by reporting a change in the treatment of data in recent works: while previous work showed that the majority of decision support systems used simple processing mechanisms to handle data collected in real-time, more recent work showed an increasing number of management systems that use complementary technologies that rely on cloud and big data computing for processing large amounts of data. In terms of research domain, this article addresses the agriculture economic sector, including indoor and outdoor agriculture (greenhouse, hydroponics, crop beds, pots, orchards, permanent crops, and arable lands). However, the livestock farming (both indoors and outdoors) was left out because, due to its specificities like impact on nature, associated mobility, different species (mammals, birds, fishing) deserves a separated and specific review.

For this purpose, the remaining work is organized as follows: Section 2 presents the introductory concepts discussed in this study, Section 3 presents the methodology followed for the systematic review, Section 4 details the results obtained, Section 5 presents the considerations and Section 6 presents the conclusions.

## 2. Concepts

To better understand the questions related to the use of IoT in agriculture during the 21st century, it is important go deeper in some fundamental concepts.

### 2.1. Smart Farming

Smart farming can be defined as the application of supplementary technologies to agricultural production techniques to help minimize waste and boost productivity.

For such, intelligent farms use technological resources that help in various stages of the production process, such as monitoring of plantations, soil management, irrigation, pest control, delivery tracking, etc. [16]. Such resources include, among others, temperature, luminosity, humidity, pressure, ground chemical concentration, unmanned flying equipment, video cameras, agricultural information management systems, global positioning systems (GPS) and communication networks [17].

The integration of technological resources into the agricultural production process is a relevant issue. From an economic point of view, the precision agriculture market is expected to have a revenue of US$10 billion in 2023 [18] with opportunities for technology providers, agricultural equipment and machinery providers, producers and others involved in this business. In addition, smart farms are expected to be able to optimize food production by improving the application of nutrients to the soil, reducing the amount of pesticides and water consumption in irrigation [19].

### 2.2. Internet of Things

IoT can be understood as a network of interconnected intelligent devices capable of communicating with each other, generating relevant data about the environment in which they operate. Thus, virtually any device capable of establishing a connection to the Internet can be considered a “thing” within the context of IoT, such as household appliances, electronics, furniture, agricultural or industrial machinery and even people [6].

Although the idea of IoT is not new, its adoption has increased in recent years, mainly thanks to the development of technologies that support it, among which the improvement of hardware—with the consequent reduction in size and power consumption—improvements in connectivity with the Internet and between devices via wireless connection, cloud computing, artificial intelligence and big data. All these technological components help build a network of devices capable of sharing data and information, as well as acting actively based on network inputs [20].

According to [21], the architecture of IoT systems is similar to the architecture of other computer systems but it must take into account the particularities of this paradigm, such as the limited computing capabilities of the devices, identification, detection and control of remote objects.

### 2.3. Intelligent Agriculture

**Figure 1:**
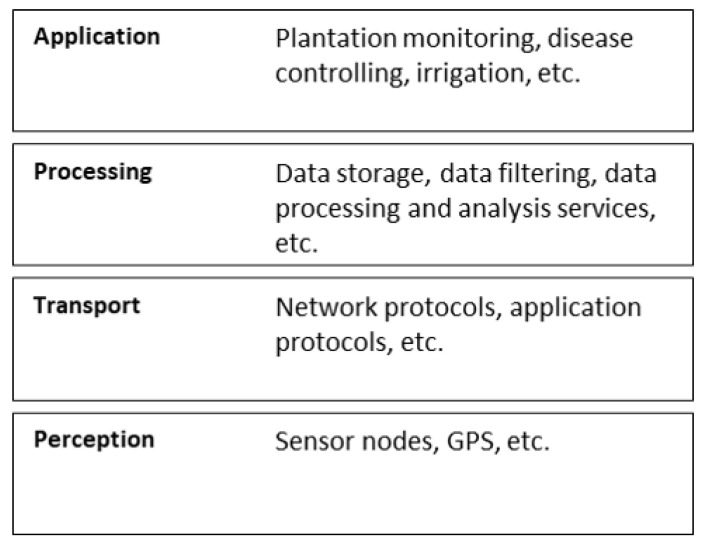
IoT solution architecture that includes 4 layers: perception, transport, processing and application, based on [22,23].

The IoT architecture proposed in [22] and [23], and shown in Figure 1, presents four layers, considering the main components of an IoT solution: devices, network, services and application.

The perception layer relates to the physical devices in the solution and how they interact with each other and with the transport layer. These devices are responsible for collecting data, enabling the communication of the so-called “things”. This can be done by using commercial solutions—such as UAV devices [24], sensor nodes [25]—or new devices, developed with components like sensors and single-board computers (SBC)—such as Arduino or Raspberry Pi—to build sensor nodes and communication gateways. Sensor nodes, for example, are used to monitor plant diseases [26], control environmental variables in greenhouses [27] and external crops [28,29,30], among others. The interaction between the devices that belong to the perception layer and the services that belongs to the processing layer is intermediated by the transport layer and might occur in several ways, such as through the direct communication between sensor nodes and a data processing platform (such as FIWARE [31], SmartFarmNet [32] and Thinger.io [33]) or through a gateway that, besides intermediating the communication between sensor nodes and the internet, acts as a data hub and enables the communication between network protocols that are originally incompatible, such as ZigBee and the Internet [28].

The transport layer refers to the network and transport capabilities such as network and application protocols [22]. IoT solutions use network protocols to enable communication between the perception layer and the processing layer. These protocols are used to create the so-called wireless sensor networks (WSN), that allows wireless communication between sensor nodes and applications. Each protocol has important characteristics, such as the data exchange rate, range and power consumption. Based on these characteristics such protocols can be classified in short-range, cellular networks and long-range [34]. Protocols for short-range networks (e.g., Bluetooth, ZigBee and Wi-Fi) enable communication in short distances. According to [34], usually such protocols have a high data transmission rate and low power consumption. Therefore, they are used for the communication between devices that are near each other. Protocols for cellular networks (e.g., GPRS, 3G) enable communication in long distances and with a high data transmission rate. However, they have a high power consumption [35] and costs for licensing [34]. Protocols for long-range networks (e.g., LoRaWAN and Sigfox) enable communication in very long distances [34]. These protocols are used to establish the low power wide area networks (LPWAN) due to the fact that they have a low power consumption [36]. However, the data transmission rate of these protocols is low. Therefore, these protocols are appropriate for use when the solution needs to transmit a few amounts of data in very long distances. Table 1 presents the characteristics of some network technologies used for IoT.

As shown in Table 1 there is a trade-off between coverage, data rate and energy consumption. Considering the technologies for star networks presented in Table 1, it is possible to notice that energy consumption is higher in technologies with a high data rate and short coverage. On the other hand, LoRa has a small data rate but a large coverage and low power consumption. These questions are especially relevant when considering agriculture because agricultural scenarios often have limited or no energy supply and obstacles for wireless communication.

Different topologies can be used for implementing networks, such as tree, star and mesh. Star networks have a central node and several peripheral nodes. The communication in such topology occurs as follows: peripheral nodes send data directly to the central node. The central node can implement capabilities for routing messages and communicating through multiple network protocols [23]. Tree networks are composed of router nodes and leaf nodes. Such networks can be understood as a cluster of star networks. Within each cluster, leaf nodes send messages their father node. In mesh networks, in theory, each node can be a router with rerouting capability. Thus, messages in mesh networks are routed hop by hop until reaching the final destination [37].

Data is sent to the destination through application protocols such as the message queueing telemetry transport (MQTT) [38] or the constrained application protocol (CoAP) [39]. MQTT is an open-source messaging protocol that enables communication between constrained devices and in unreliable networks [40]. MQTT runs over TPC/IP or similar protocols (e.g., Bluetooth) [41], which makes the use of MQTT appropriate for different IoT solutions. The MQTT protocol, which is based on the publish/subscribe architecture, allows communication between devices to take place in the following way. First, devices publish messages that are structured in topics on a message broker. Then, other devices read these messages by subscribing to relevant topics on the message broker. These topics allow the organization of messages based on categories, subjects, etc. [42]. The use of MQTT for communication between device allows low coupling between the device that publishes the message and the devices that listen to the messages, the so-called “one-to-many” communication [38]. Like MQTT, CoAP is a communication protocol optimized for constrained devices and unreliable networks. However, CoAP messages are interchanged using User Datagram Protocol (UDP) and the CoAP protocol is based on the client/server architecture. This architecture requires that a connection is established between devices before any messages are transmitted [38]. For this reason, communication using CoAP works in the following way. First, the device that sends messages needs to know the address of each device that is expected to receive messages. Then, messages are sent over UDP to the specified address. Due to the use of UDP, CoAP messages are classified accordingly to the required status of confirmation of receival, for example, confirmable or non-confirmable [39]. The CoAP protocol does not implement a structure of topics for messages. However, a similar approach can be implemented using application programming interface (API). Nonetheless, the use of CoAP creates a high coupling between the device that sends messages and the device that is expected to receive messages, as the communication is “one-to-one” [39].

The processing layer comprises data storage, visualization and processing resources. In this context, big data allows distributed storage and parallel data processing, enabling the extraction of information in the shortest possible time [43]. Such information are used as models by artificial intelligence (AI) systems—which, according to [44], can be understood as the ability of a system to operate as if it had the thinking capacity of a human being—and machine learning—that, according to [45] is a data processing technique to detect patterns and correlation among complex and unrelated data—for the development of decision support systems and automation of irrigation control systems [46], monitoring [47] and diseases detection in crops [48], for example.

Finally, the application layer comprises IoT applications that, supported by the other mentioned layers, provide management information to farmers, being able to manage the entire production process in the plantations.

## 3. Methods

Several related works are being developed in recent years. This rich literature has already been analyzed by the academia from multiple perspectives with objective of determining the state of the smart farming development. Thus [49] presented a systematic review of precision livestock farming in the poultry sector and [50] made a review of state of the art of technologies used in precision agriculture, focusing in the innovations, measured parameters, technologies and application areas. On the other hand [4] has focused on the use of big data as a tool to support agriculture, pointing out the main opportunities and challenges of using this technology. Finally, [51] presented a quantitative literature review on smart farming related papers, helping to outline an overview of academic production related to the subject. In this way, the present work aims to complement such analyses by making a systematic review of IoT solutions applied to smart farming.

To reach the proposed objectives, this study has used the Preferred Reporting Items for Systematic Reviews (PRISMA) methodology, which is a framework developed to support reports and systematic reviews of literature [52].

As a research strategy, in October 2019 a search was made in the Scopus database through the search tool available on the website. In addition, in June 2020 a new search was made in the same database to include papers published in 2020. The choice of this database took into consideration its scope and relevance in the academia, since this database indexes several journals and catalogues, such as IEEE, ACM and Elsevier, besides being widely used in similar bibliographic reviews, as in [4] and [51]. In addition, in February 2020 a new search was performed in the same database. The strategy adopted for the work research in this database looked for terms used to refer to the application of technology in the area of agriculture, such as “Precision Agriculture”, “Precision Farming”, “Smart Farming” and “Smart Agriculture” in association with “IoT” and synonyms terms. The publication date of the articles was not a criterion for ignoring them. The scope of the research was limited to documents such as journal and conference articles, published in English, Portuguese or Spanish, and whose access was fully available. Thus, the resulting search instruction for the database was as follows:

(“Smart Farming” OR “Smart Agriculture” OR “Precision Farming” OR “Precision Agriculture”) AND (“IoT” OR “Internet of Things” OR “internet-of-things”) AND (LIMIT-TO(ACCESSTYPE(OA)))

It should be noted that the quotation marks have the function of ensuring that terms composed of multiple words were searched together, thus preventing words from being considered individually.

After extracting the articles that resulted from the search, they were manually reviewed through the analysis of the title, keywords, abstract and text. Initially, based on this review, the works identified in the researched database were consolidated, thus eliminating duplicate articles.

Subsequently, the articles were validated as to their framing in the objectives proposed for this study and considered valid when: (i) they were not a review or bibliographical research (ii) they were related to theme (iii) they presented a technology or solution based on IoT to solve problems related to agriculture (iv) they were published in English, Portuguese or Spanish. Furthermore, works were also excluded when they were related to livestock activities instead of agriculture.

**Figure 2:**
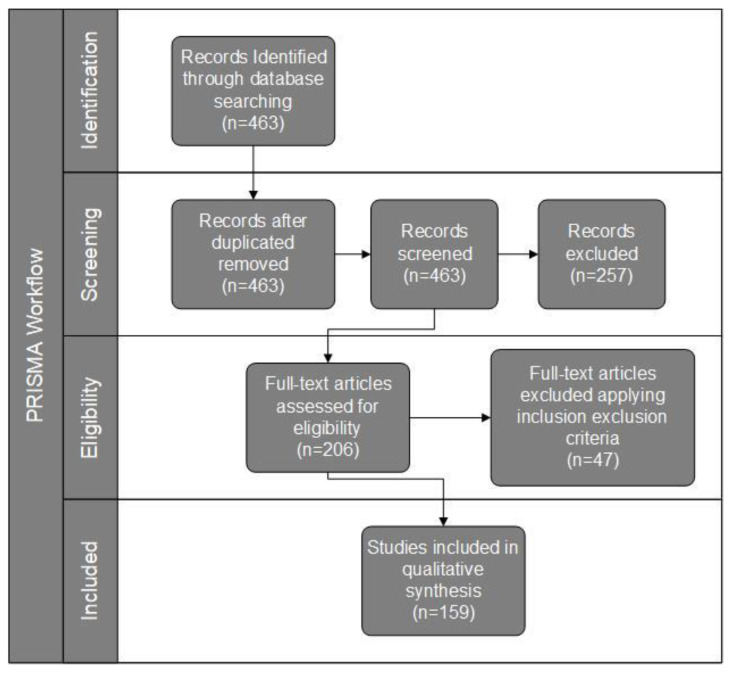
PRISMA flowchart of the systematic review on state-of-the-art IoT solutions in smart farming.

The process of searching and selecting papers for this study followed the workflow summarized in Figure 2, where it can be observed that the initial search resulted in a total of 463 articles, which were analyzed, filtered and classified in a narrowing process that culminated in the selection of 159 articles.

In the identification phase 463 articles were selected with the search tool.

During the screening phase, a manual review of the articles was carried out to identify in the titles, abstract and key words the papers adherent to the objectives proposed for this study, following the criteria mentioned in this section. Among these, 257 were considered invalid and discarded. About 62% of the discarded items did not consider smart farming to be the focus of the work, although some presented improvements for IoT that could benefit smart farming indirectly. Additionally, almost 31% of the discarded papers were studies or literature reviews related to smart farming and the use of various technologies. A smaller number of papers related to smart farming but not addressing IoT (about 5%) and papers where the abstract or text were not available (about 2%) were also discarded.

During the eligibility phase, the content of the 206 resulting articles were reviewed and the papers were classified using the same criteria used in the previous step. In this phase 47 articles were discarded. Among the discarded articles 29% were not related to IoT and 30% were not related to smart farming. The other 41% of the discarded papers were paper reviews or papers without content available. This analysis resulted in 159 articles considered eligible which were included as a sample for this study.

## 4. Discussion

**Figure 3:**
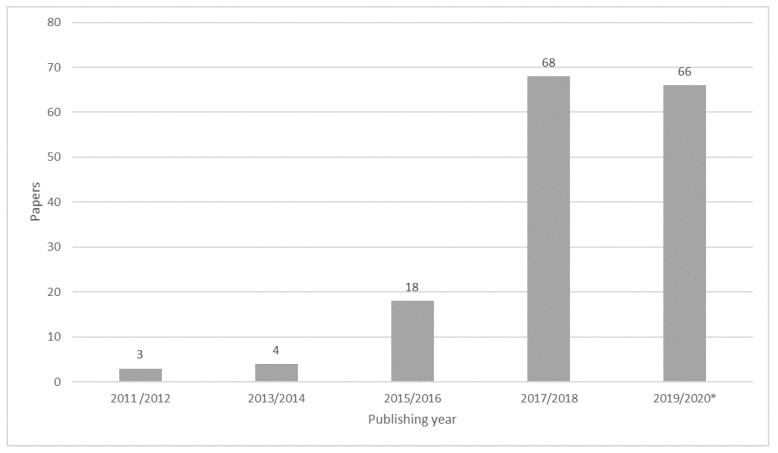
Classification of reviewed papers according to the year of publication.

Based on the results obtained in the analysis of the articles considered for this study, it was possible to observe a growth trend in the number of publications related to IoT and smart farming since 2011, with special emphasis from 2016 onwards, as shown in Figure 3.

It is possible to observe an expressive increase of 278% in the number of published papers in 2017/2018. It’s also possible to observe a very similar number of published papers in 2019/2020, until the first semester of 2020. The amount of published papers in recent years evidences the increasing in discussion and the relevancy of the topic IoT applied to smart farming.

**Figure 4:**
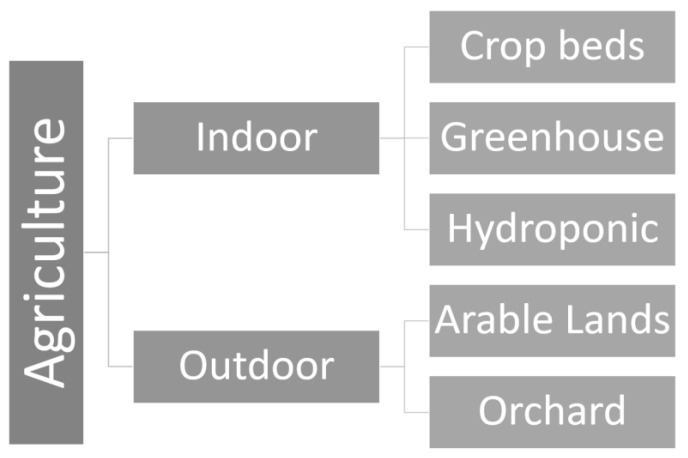
Typical scenarios in agriculture can be divided in indoor and outdoor. Indoor agriculture includes environments such as greenhouse, hydroponics and crop beds. Outdoor agriculture includes environments such as orchards and arable lands. IoT solutions that may be applied to multiple environments are referred to as “Generic”.

Within the reviewed papers it was identified the main scenarios and environments of agriculture. As shown in Figure 4, such scenarios can be divided into indoor and outdoor. Environments for indoor scenario are protected from climatic impacts, such as solar radiation, rain and wind. Examples of environments for indoor scenarios include greenhouse, hydroponics, crop beds, pots, etc. In contrast, environments for outdoor scenario are more susceptible to climatic impacts. Examples of environments for outdoor scenario are arable lands, orchards and generic outdoor plantation.

### 4.1. Application

Within the reviewed papers it was also identified that the most common applications of IoT solutions for smart farming are:

- Chemical control (e.g., pesticides and fertilizers).
- Crop monitoring.
- Disease prevention.
- Irrigation control.
- Soil management.
- Supply chain traceability
- Vehicles and machinery control.

Table 2 presents the reviewed papers, grouped by agricultural environment and application of the IoT solution. It is worth mentioning that several IoT solutions presented on the reviewed papers could be applied to multiple environments (Figure 4). Thus, such IoT solutions are classified as “Generic”. Additionally, the “Others” column in Table 2 includes papers whose IoT solutions were developed for agricultural environments that were less mentioned, such as pots, crop beds, etc. It is possible noting a predominance in projects where the application is for crop monitoring, irrigation management, and disease prevention.

As shown in Table 2, the most common application of IoT solutions for smart farming is crop monitoring. Moreover, as shown in Table 2, these solutions have been developed for multiple agricultural environments, such as arable lands, orchards, greenhouses, etc. The fact that this type of application is so common in agriculture can be justified by the relevance that crop monitoring has for farmers. IoT solutions developed for monitoring crops focused on collecting environmental data of plantations (such as temperature, humidity, luminosity, etc.). Farmers can use these data to obtain a better insight of the plantations. For example, such data was used to determine the vigor of rice [47,58], alfalfa [30] and maize [57] crops and to control the environmental conditions of greenhouses [99,100,102,104]. Similarly, IoT solutions for irrigation control has also been developed for multiple agricultural environments, as demonstrated in Table 2. Such IoT solutions aimed to optimize the use of water resources in agriculture in different ways, such as by simply using sensors for measuring the soil moisture and using these data for controlling the irrigation source [34,139] or in a more sophisticated way, by combining humidity data with datasets of weather to determine the amount of water required during the irrigation [140]. IoT solutions for disease prevention aimed to identify and prevent diseases on plantations. For this purpose, these IoT solutions collected multiple environmental and plantation data, such as images of plants [129,132,134], sounds [135], temperature, humidity, etc. [131,137]. These data were processed with different approaches, such as image processing [129,134] or artificial intelligence [132,137]. For example, the IoT solution developed in [129] processes images collected from a sugarcane crop and identifies diseases on the leaves of plants. In addition, [135] developed an IoT-enabled device that captures sounds produced by larvae inside trees. IoT solutions for chemical control presented in Table 2 aimed to optimize the application of fertilizers and pesticides on plantations. For this purpose, these IoT solutions collect data (such as nitrogen, salinity or PH) from the crops. Based on the collected data, such IoT solutions can identify crop areas that may require the application of fertilizers or pesticides. For example, in [54] aerial images of crops are processed to determine the nitrogen concentration in a large plantation. These images are useful to determine the specific region that requires fertilizer. In addition, [55] developed an automated robot that optimizes the application of pesticides in greenhouse cultivations. IoT solutions for soil management aimed to identify different soil attributes used for planting. For example, such IoT solutions are used to measure the soil moisture [163], to identify the water consumption pattern [159,161] and to identify the nutrients of the soil [158]. IoT solutions for vehicles and machinery control focused on collecting data of and managing agricultural equipment and machinery such as tractors, harvesters and trucks. For this purpose, IoT solutions had to deal with the characteristics inherent to agricultural equipment, such as mobility. Data from the equipment itself, such as implement status, engine performance, or speed are collected using sensors [41] to optimize their maintenance cycle. Additionally, due to the mobility of agricultural equipment, opportunistic computing was used to collect data from remote crop areas by using sensors coupled to tractors [174].

Each agricultural environment presented in Table 2 brings its own challenges for the projects, which includes the environment impact on the communication between sensors, either by the distance between the sensor nodes [25,105,180], by the lack of communication in the croplands [98,174] or even by the impact of vegetation in the signal propagation [70,175]. Furthermore, as indicated in [28], climatic elements—such as rain, snow or solar radiation—have influence on both the planting and the sensor nodes.

To cover these scenarios commercial electronic sensors are used by 96% of the reviewed papers. This expressive usage can be justified by the fact that such sensors are affordable, certified, ready-to-market and meet the main monitoring needs in IoT solutions for smart farming. Such sensors are used for collecting real-time data about multiple agricultural parameters, such as climatic data, substrate information, luminosity, CO2 concentration and images through cameras and multispectral sensors, as shown in Table 3. Moreover, several papers (4%) focused on developing custom-made sensors for monitoring specific agricultural aspects, such as soil nutrients (e.g., nitrate [158]) and leaf evapotranspiration for measuring the hydric stress in tobacco crops [81].

Different types of sensors are used in agriculture for collecting data from different aspects of agriculture such as crop monitoring, substrate monitoring and environment monitoring.

As presented in Table 3, different types of sensors were used in IoT solutions for smart agriculture to collect data from multiple aspects of agriculture, such as the crop, substrate, environment and other. For this purpose, as shown in Table 3, for environment monitoring electronic sensors were used in IoT solutions to collect environmental data, such as temperature, humidity and luminosity [104,109,114]. In addition, for substrate monitoring electronic sensors were used to collect data from the substrate (e.g., soil and water), such as temperature, moisture and nitrogen. Likewise, pH sensors were used for measuring the acidity or the alkalinity of the water in hydroponics cultivations. For crop monitoring, cameras and multispectral sensors were used to collect images of crops. These sensors can be installed on an UAV to obtain aerial images of large plantations [47,57,58] or used in robots to retrieve a detailed image of the leaf of a plant [111].

### 4.2. Perception

The choice of hardware is a very important aspect of the IoT project development because it impacts the costs and the technologies that can be used. 60% of the reviewed papers mentioned the hardware used to support the IoT solution. Furthermore, SBCs were mentioned by 40% of the reviewed papers. The use of SBCs can be justified by the fact that these devices are affordable and versatile [38], enabling the development of custom-made IoT devices. For example, some SBCs such as Arduino has an integrated development environment (IDE). This IDE enables the development of custom programs to be installed as firmware on the Arduino boards [184]. Similarly, Raspberry Pi is compatible with several operating systems, such as Raspbian, Ubuntu Core or Mozilla Web Things [185]. Some of these operating systems are open-source, which allow for the customization of its source-code. Besides, these operating systems support applications developed with programming languages such as Python [26]. Furthermore, the capabilities of SBCs can be extended by associating them with other hardware components, such as sensors or transceivers. This characteristic makes SBCs able to work as gateways or sensor nodes in IoT solutions. Among the papers that mentioned SBCs, 82% mentioned the use of Arduino, Raspberry Pi and ESP boards (such as ESP8266, ESP12 and ESP32). Table 4 presents the application of embedded system platforms and UAV devices in smart farming.

As shown in Table 4, IoT-enabling devices are used for multiple applications on IoT solutions for smart farming. SBCs were used both as sensor nodes and gateways. Table 4 reveals that Arduino was the most commonly used embedded system platform among the reviewed papers. The extensive use of Arduino can be justified by the fact that Arduino is open-source hardware that enables the development of different devices through the use of boards that extend their native functionality. Table 4 also shows that embedded system platforms have been more widely used in IoT solutions for crop monitoring. As sensor nodes, for example, in [124] sensors for collecting environmental data such as soil humidity, solar radiation and rain are connected to an Arduino Uno. The Arduino is, then, used to monitor the health of a vineyard. Likewise, in [117] a Raspberry Pi is used to manage the temperature and air humidity of a greenhouse. IoT devices are also used as gateways to connect short-range WSN with the internet by using long-range communication protocols. For example, in [127] a gateway is used to connect WSNs using 3 different protocols (ZigBee, Bluetooth and Wi-Fi) with a remote server by using 3G. In [84] a LoRaWAN gateway obtains data from sensor nodes using LoRa and retransmits this data to a cloud-hosted platform by using 4G. 3G and 4G are cellular network technologies that, as discussed in Section 2.3, enable communication in long distances and with a high data transmission rate. These technologies will be discussed with more details in Section 4.3.

In addition, Table 4 also reveals that UAV is widely used by IoT solutions for monitoring crops, disease prevention and chemical control. The use of UAV for crop monitoring is due to the fact that UAV has the potential to accelerate and reduce the cost of monitoring extensive crops. For this purpose, cameras and multispectral sensors are attached to UAV devices that are used to obtain aerial images from large crops. Such images are processed by the IoT solution to calculate agricultural parameters, such as the leaf area index (LAI). The LAI is a parameter used to determine the vegetation coverage within a specific area. LAI, combined with other parameters, can be used to evaluate the amount of nitrogen in rice crops [58], determine the vigor of rice and maize [47,57] crops and detect diseases in sugarcane crops [129]. Moreover, UAV devices are used in [46] to optimize the application of pesticides and fertilizers in arable lands.

### 4.3. Network

Data obtained with sensor nodes are usually sent to the destination (e.g., database, server, IoT platform) through a wired or wireless network. Within the reviewed papers, 60% have mentioned the network protocol used in the IoT solution. Among the mentioned network protocols, CAN and Ethernet were the most used ones for wired networks. Likewise, LoRaWAN and protocols for cellular network (e.g., GPRS, 3G, etc.) were the most used protocols for long-range wireless networks. Analogously, ZigBee, Wi-Fi and Bluetooth were the most used protocols for short and mid-range wireless networks. Table 5 shows network protocols used for the IoT solutions within the reviewed articles.

As shown in Table 5, several network protocols are used in different environments of agriculture (e.g., arable land, greenhouse, orchard) to enable communication between IoT solution devices, such as sensor nodes and gateways. Such network protocols enable the creation of short or long-range networks. Table 5 reveals that for short and middle-range communication, IoT solutions of the reviewed papers used different technologies, such as Wi-Fi, ZigBee and Bluetooth. Moreover, it is possible to observe in Table 5 that Wi-Fi is the most common network technology for communication within the analyzed articles. This extensive use of Wi-Fi can be justified by the fact that Wi-Fi is a ubiquitous technology and, therefore, easy to implement. However, due to the higher energy consumption of Wi-Fi, low-energy consumption technologies, such as ZigBee or Bluetooth, are also extensively used. For example, [62] used ZigBee to send images from a plantation to a remote server and [188] developed a sensor node that uses Bluetooth to deliver monitoring information from the farm directly to an application installed on a smartphone. Table 5 also demonstrates that IoT solutions of the reviewed papers used cellular networks, Sigfox, or LoRaWAN for long-range networks. Cellular networks are prevalent in IoT solutions for Smart Farming. This can be justified by the fact that cellular networks allow the communication of IoT devices in long distances and with a high data rate. For example, [141] uses cellular network to send data collected from humidity sensors to a cloud-based platform and to control an irrigation system. Similarly, Sigfox and LoRaWAN enable communication in very long distances while requiring low energy to operate. Based on these characteristics, Sigfox and LoRaWAN were used for long-range communication, as an alternative to cellular networks or in regions where there was no cellular network coverage. Sigfox is used in [34] as the network protocol of an IoT solution used to control the irrigation of a plantation. Likewise, in [137] the LoRaWAN is used to send data from multiple sensors installed in a greenhouse to a remote platform.

Besides the distance between sensor nodes, gateways, and other network elements, the vegetation itself can be an obstacle for sensor communication, as demonstrated by [175] and [70] who analyzed the impacts on signal propagation on 433 MHz and 2.4 GHz frequencies in rice plantations and an orchard. An additional challenge for greenhouses arises from the high density of sensors, which can lead to interference in the wireless signal due to proximity [105,112,180]. To mitigate this problem wired networks, such as CAN [100] or Ethernet [122], can be used. As shown in Table 5, these technologies have been more used in greenhouses, because usually this type of agricultural environment is more appropriated for implementing wired networks. Moreover, [112] investigated the path loss on wireless signals and concluded that the proper positioning of directional antennas can optimize the number of sensory nodes required for monitoring a greenhouse.

Network topology is another important aspect of an IoT solution. According to [61] the topology of sensor networks can be star, tree (or cluster) or mesh. The network topology impacts the distance between the sensor nodes and the destination and, consequently, the number of sensor nodes in the WSN [190]. For example, star networks are composed of a central node (coordinator) and several peripheral nodes. In such topology, peripheral nodes send data to the central node [93]. Therefore, the maximum distance between the peripheral nodes and the central node is limited by the maximum distance allowed by the physical layer communication standard. On the other hand, as discussed in Section 2.3, in mesh networks each node has routing capability, hence extending the network coverage by allowing multi-hop communications [191]. Based on the architecture of the IoT solution and on the project description it was possible to identify the topology adopted by 61% of the reviewed papers. For example, a star topology is used in [34] for connecting sensor nodes to a central node using the LoRa protocol. This central node acts as a gateway and retransmits messages to a cloud-based application that controls an irrigation system using Sigfox. Also, in [107] the star topology is used to connect multiple sensors within a greenhouse. Such sensors use the ZigBee protocol to send messages to a central node, which acts as the network gateway. Mesh networks are considered more complex to be implemented but also more reliable due to the redundancy of communication between the sensor nodes [105]. Such topology is used in [105,107] for monitoring a greenhouse. Tree (or cluster) networks combine multiple star networks. Both [61] and [178] implement a cluster network for monitoring crops. In [61] sensor nodes collect information from a crop and send messages to a router node. This router node acts as the gateway of the cluster and retransmits the message to the main router node of the network. In [178] several router nodes are deployed in the crop area in order to optimize the energy consumption of sensor nodes.

**Figure 5:**
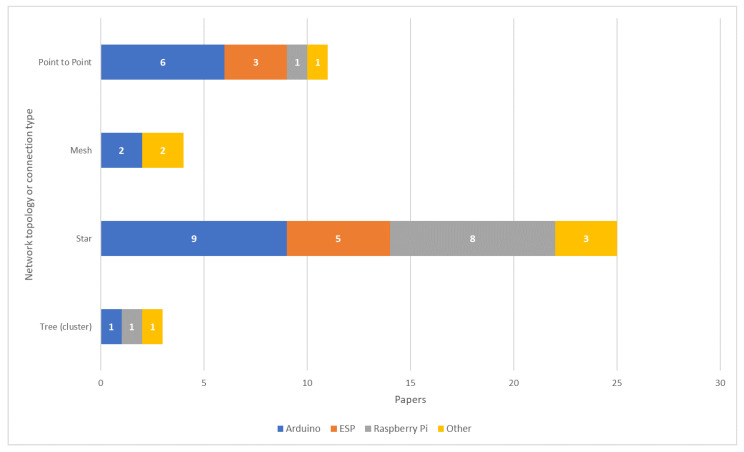
Distribution of IoT-enabling devices by network topology or device connection type within the reviewed papers.

Furthermore, embedded system platforms have been used to support network topologies. The chart in Figure 5 presents the distribution of embedded system platforms by network topology or device connection type. It is worth mentioning that although point to point is not a network topology, this type of device connection was used in several IoT solutions within the review articles. As shown in Figure 5, Raspberry Pi is often used in IoT solutions implementing the star network topology. Arduino is the embedded system platform used in multiple types of network topology or device connections. Additionally, Arduino is the most frequently used embedded system platform to support star network topology and point-to-point communication. Finally, ESP-based devices include devices that use system-on-a-chip (SoC) modules such as ESP-32 and ESP8266 (Espressif Systems, Shanghai, China). ESP-based devices are often used in IoT solutions that implement star network topology or point-to-point communication.

As mentioned in Section 4.2, embedded system platforms can be used to build gateways or sensor nodes. As shown in Figure 5 the use of Raspberry Pi, Arduino and ESP stand out, probably because such embedded system platforms are cost-effective [38] and enable different network protocols (e.g., ZigBee, Wi-Fi and Bluetooth) with the use of transceivers. This characteristic allows such embedded system platforms to act as sub-nodes and central nodes in a star network [27,30,34] or as router nodes in mesh and cluster networks [106,178].

IoT devices transmit information to cloud-based platforms or applications through application protocols [109]. Such protocols can follow the publisher/subscriber architecture which, as mentioned in Section 2, are appropriate for devices with limited computing resources. Among the application protocols used in the reviewed papers HTTP, MQTT and CoAP stand out. Such application protocols are useful to enable compatibility between non-standardized IoT devices and IoT platforms. For example, SmarFarmNet developed in [32] adopts the “bring your own IoT device” concept by implementing loosely coupled application protocols such as MQTT and CoAP. Furthermore, although HTTP is not a specific protocol for machine-to-machine (M2M) communication, its use associated with REST APIs enables low coupling between IoT devices and applications, analogous to MQTT, for example. However, as [109] concludes, the MQTT protocol is preferable for smart farming applications due to its resiliency, interoperability across different network protocols and transmission rate.

Finally, although the power consumption is not an exclusive topic within the transport layer, according to [178] the highest power consumption for IoT devices within a WSN occur during the transmission of data. This review identified several approaches for optimizing the power consumption in IoT solutions for smart farming. Among the identified solutions are the use of low energy protocols (e.g., BLE, ZigBee, Sigfox), reduction of data transmission in sensor nodes by an optimized duty cycle [177,178,192] and the use of message routing approaches that are more energy-efficient [72,193].

### 4.4. Processing

**Figure 6:**
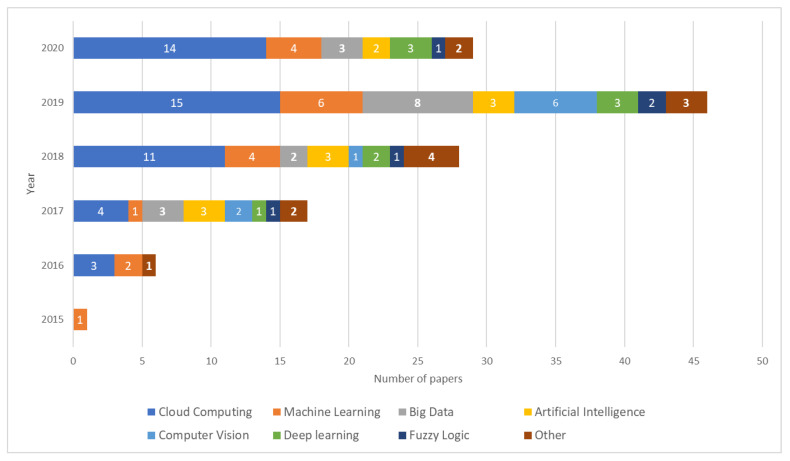
Techniques and technologies for data-processing in smart farming identified within the reviewed papers. In recent years, the use of modern processing techniques, such as artificial intelligence and big data became more common. IoT applications relies on cloud computing for storing and processing the big data of agricultural information collected by IoT devices.

Among the analyzed papers it was possible to observe that initially, the main objective of IoT solutions was to collect and store data from sensor nodes. However, in more recent years, it is possible to observe an increasing number of IoT solutions that used supplementary techniques and technologies to treat the collected data, such as cloud computing and big data. Likewise, it is possible to observe an increasing number of works that used simultaneously two or more techniques or technologies for processing data. As shown in Figure 6, the most cited technologies within the reviewed papers are cloud computing (34%), machine learning (15%), big data (13%), and artificial intelligence (9%).

Table 6 presents IoT solutions that relied on cloud-based platforms for processing data and highlights the main data processing techniques (e.g., Artificial Intelligence, Big Data, etc.). The column “Other/Not identified” comprehends IoT solutions that have used cloud-based platforms but have either (i) used any of the data processing technologies identified by other columns on Table 6 or (ii) not explicitly mentioned the type of data processing technology that was adopted.

Table 6 reveals that the most found cloud-based platforms in the reviewed papers are ThinkgSpeak, FIWARE, Ubidots, SmartFarmNet, AWS IoT and Thinger.io. In particular ThingSpeak is the most used cloud-based platform across all the reviewed papers, due to the fact that this platform is open-source with low infrastructure requirements [34]. In addition, Table 6 shows that AWS IoT was used with a higher number of data processing techniques. Not all cloud-based platforms offer the same set of functionalities, but in general, they have capabilities for data storage [30,94,102,122,132], processing [194] and visualization [102] and action control on farms [34]. Furthermore, Table 6 also reveals that, even though there are multiple cloud-based platforms, several reviewed papers developed their own cloud-based platform for the IoT solution.

Cloud-based platforms provide scalability for IoT solutions by relying on cloud computing to process and data. For instance, some platforms shown in Table 6, such as Thinger.io [33], are built entirely on top of infrastructure services provided by cloud providers (e.g., Amazon AWS and Microsoft Azure). Also supported by such services, the platforms make available data analysis modules with graphics and panels that allow real-time monitoring of the information obtained or the creation of customized panels from the integration of multiple data [33].

Due to the scalability provided by these platforms, the large amount of data generated by the sensors is stored in databases to form the so-called big data, an unstructured set of information that is used to generate information about crops. According to [197] big data demands the use of technologies to optimize the processing time due to the large volume of information. For example, Hadoop—a parallel database for big data applications—proved to be efficient when analyzing the rainfall index data from several meteorological stations [197].

IoT solutions use different types of techniques and technologies for processing the collected data. Table 7 presents commonly used technologies per applications as identified in the reviewed papers. Column “Other Technologies” encompasses all the technologies that are not identified by any of the other columns in Table 7.

Table 7 reveals that the most commonly used technologies to support data processing are artificial intelligence, machine learning, and big data. The use of these technologies is related to their ability to process large amounts of information in a short time. In addition, Table 7 also shows that crop monitoring is the most common type of application for IoT solutions that have used data processing technologies. Moreover, crop monitoring is also the type of application that used the most different technologies for data processing. This can be understood by the fact that usually IoT solutions for monitoring crops collect a bunch of data and rely on machine learning and big data to process such data.

As demonstrated in Table 7, bigdata was used for different applications in IoT solutions, such as crop monitoring, soil management and irrigation control. For example, supported by big data, in [142,161,194] the soil moisture data gathered by physical sensors were related to data made available in datasets, such as the NASA Prediction of Worldwide Energy Resources (POWER) [198]—which contains meteorological data—purchase and sale values of crops, information from the user and government agencies to optimize the amount of water in irrigation cycles, support the farmer in the acquisition of agricultural inputs—such as seeds and fertilizers—and generate information and perspectives about other activities related to agriculture. Big data was also used by [56] in the development of a decision support system to provide irrigation and monitoring advice to farmers from a knowledge base created with data obtained by physical sensors (e.g., temperature, soil moisture) and virtual sensors (e.g., soil type, season). Virtual sensor is a type of software that, given the available information, processes what a physical sensor otherwise would [199].

In addition, automatic management with IoT depends on the manipulation of multiple variables. Initially, the simple observation of soil humidity and temperature can be used to trigger irrigation or cooling systems, as proposed by [187]. Nevertheless, greenhouse management can be more complex. As shown in [104,107,120], greenhouse parameters like temperature and humidity are closely tied and changing one of them can affect several others.

Fuzzy logic, as indicated in Table 7, was used in IoT solutions applications that need to handle multiple variables, such as irrigation control and monitoring crops. For this purpose, [120] uses fuzzy logic to handle multiple variables of temperature and humidity into a greenhouse and determine when a cooling system and an irrigation system should be started. Similarly, [73] uses fuzzy logic to optimize the number of sensors for monitoring soil temperature and moisture. Machine learning was also used in data processing by [46] to predict environmental conditions based on the forecast values of weather, humidity, temperature and water level and thus to control an irrigation system, by [47] to combine multiple parameters obtained from images, such as color and texture indices and by [48] to identify marks on the plants and, thus, to identify possible diseases. Similarly, in [58,125] it was used to detect diseases, identify growth stages and the health of plantations.

Similarly, as shown in Table 7, IoT solutions used computer vision for applications that need to deal with image processing, such as crop monitoring and diseases prevention. It was also possible to observe in the reviewed papers the use of computer vision to identify and classify elements in images obtained by cameras, enabling the identification of fruit in an orchard [200] or the existence of diseases and pests in plantations [48,129,133]. Additionally, in [133] computer vision was used as a monitoring tool to detect the presence of insects that can cause diseases in olive groves and in [48] the same technique was employed to analyze diseases that cause morphological deformations in plants. Additionally, computer vision was used in crop management systems, for example in [134] where it was implanted in a robot equipped with a camera and other sensors, being able to obtain images of vegetation and, through computer vision, detect weeds in plantations and eliminate them. Similarly, in [111] a robot can identify a plant and interact with the environment to irrigate it, if necessary.

Finally, blockchain proved to be an opportune technology for systems that need to implement traceability of the supply chain, as shown in Table 7. According to [171] blockchain is a global public distributed ledger that records all transactions between users. In fact, this type of control is relevant for agriculture in several aspects, such as food safety, guarantee of origin or cost reduction. To ensure information security, this technology was proposed by [30,167,171] for agricultural product traceability. For example, in [171] an IoT solution uses blockchain to record information regarding the tea production based on 5 business processes: production plan, quality inspection, sales processing, product quality inspection and order delivery. In [167] a production tracking system for agricultural cooperatives have been developed. In [30] a similar system is being proposed but still in development stage.

## 5. Considerations

IoT solutions for smart farming take advantage of the scalability provided by platforms and cloud computing to store large amounts of data obtained by sensors. These big data of specific information may be processed with artificial intelligence techniques—such as machine learning—to improve the management of smart farming. For example, the processing of big data may be used to obtain crop insights, optimize water resources and increase the crop quality by preventing disease and reducing the amount of chemical products employed. Crop monitoring solutions use SBC (e.g., Arduino and Raspberry Pi) or UAV (e.g., drones) together with sensors (e.g., humidity, temperature, CO_2_ or image) to collect data in indoor or outdoor environments.

Different types of network connections are used for communication between IoT devices, such as wired and wireless connections. The review shows that wired networks, such as CAN and Ethernet, are used for indoor agriculture (e.g., greenhouses). The use of wired network on indoor agriculture may be justified by the fact that in this scenario the physical components of the network are less susceptible to climatic agents impacts. Likewise, generally distance between sensor nodes in indoor agriculture enables this type of connection. Wireless connection, on the other hand, is used both in indoor and outdoor agriculture. Wi-Fi is the most mentioned protocol within the analyzed projects, due to its ubiquitous utilization in the daily life. However, power consumption and signal range characteristics may limit use of Wi-Fi in larger projects or in projects with power restrictions. To overcome the power consumption issue, energy-efficient protocols such as ZigBee, BLE or LoRa are used for communication in wireless networks.

Furthermore, it is worth mentioning that this review investigated papers where the IoT solution for smart farming was applied to agriculture only. However, the use of IoT for smart farming can also be applied to other activities related to farming, such as livestock [201]. Moreover, despite the fact that power-supply in IoT solutions for smart farming does not represent a specific layer of an IoT solution architecture [17,18], this topic has been covered in some of the reviewed papers. For example, [72,177] proposed improvements in algorithms for message routing and in duty cycles in sensor nodes. These approaches contribute to the reduction of power consumption by IoT devices. Similarly, a mission-based approach was used in [53] to optimize the power consumption in UAV. This approach was used to identify the most efficient path for a set of drones. Likewise, [177] proposed an intelligent activity cycle to improve the performance of data aggregators in terms of energy efficiency on cloudy days.

## 6. Conclusions

This work presented a systematic review of the state-of-the-art of IoT adoption in smart agriculture and identified the main components and applicability of IoT solutions. This review reported a change in the treatment of data in recent works: while previous work showed that the majority of decision support systems used simple processing mechanisms to handle data collected in real-time, more recent work showed an increasing number of management systems that use complementary technologies that rely on cloud and big data computing for processing large amounts of data. Furthermore, it was observed in this review that in recent work the use of artificial intelligence and image processing techniques has become more common to improve the management of smart farming. From the identified applications of IoT for smart farming it was observed that the most common application is the monitoring of crops. This review also showed that different network protocols may be simultaneously used in IoT solutions for smart farming. In addition, the comparison of types of network connections used in IoT solutions for smart farming revealed that wired networks are used in indoor scenarios (e.g., greenhouse) while wireless networks are used both in indoor and outdoor scenarios (e.g., arable lands, orchards). Moreover, the review discussed in this work suggests the increasing relevance of IoT solutions for smart farming. Future work may extend this review by including other relevant articles and complementary analysis of project costs, usability and regional challenges intrinsic to IoT applications. Another important future research direction could be the analysis of the edge and fog computing usage in smart agriculture as a way to deal with challenges associated with traditional centralized cloud solutions such as high communication latencies, lack of support for real-time reaction to detected events, large bandwidths, etc.

## Figures and Tables

**Table 1:** Examples of network technologies used in IoT [34].

Parameter	Wi-Fi	Bluetooth	ZigBee	LoRa
Standard	IEEE 802.11 a, b, g, n	802.15.1	802.15.4	802.15.4 g
Frequency	2.4 GHz	2.4 GHz	868/915 MHz, 2.4 GHz	133/868/915 MHz
Data rate	2–54 Mbps	1–24 Mbps	20–250 kbps	0.3–50 kbps
Transmission Range	20–100 m	8–10 m	10–20 m	>500 m
Topology	Star	Star	Tree, star, mesh	Star
Power Consumption	High	Medium	Low	Very Low
Cost	Low	Low	Low	Low

**Table 2:** Smart farming, application and environments.

Application	Arable Land	Generic	Greenhouse	Orchard	Other
Chemical control	[53,54]	[48]	[55]	[56]	
Crop monitoring	[24,25,30,38,47,57,58,59,60,61,62,63,64,65,66,67,68,69,70,71,72,73,74,75,76,77,78]	[29,33,79,80,81,82,83,84,85,86,87,88,89,90,91,92,93,94,95,96,97,98]	[99,100,101,102,103,104,105,106,107,108,109,110,111,112,113,114,115,116,117,118,119,120,121,122]	[123,124,125,126]	[127,128]
Disease Prevention	[129,130,131,132]	[74,133,134,135,136]	[137]		[138]
Irrigation control	[28,139,140,141,142,143,144]	[27,46,145,146,147,148]	[149,150]	[34,151]	[152,153,154,155]
Soil Management	[156]	[157,158,159,160]		[161,162]	[163]
Supply chain traceability		[164,165]	[166]		[167,168,169,170,171]
Vehicles and machinery control					[41,172,173,174]
Other	[175,176]	[177,178,179]	[180]	[181]	[32,182,183]

**Table 3:** Types of physical sensors and use in smart farming.

Use in Agriculture	Application of Sensors	Examples of Sensors (Models)	References
Crop monitoring	Growth	Cyber-shot DSC-QX100 (Sony Electronics Inc., Tokyo, Japan), Parrot Sequoia (MicaSense Inc., Seattle, WA, USA)	[57,125,126]
	Insects and disease detection	FLIR Blackfly 23S6C (FLIR Systems, Wilsonville, OR, USA)	[74,132,134]
	Active canopy sensor	ACS-430, ACS-470 (Holland Scientific, Inc., Lincoln, NE, USA)	[47,54,58]
Substrate monitoring	Soil temperature, soil moisture	DS18B20 (Maxim Integrated, San Jose, CA, USA), VH400 (Vegetronix, Salt Lake City, UT, USA), HL-69, ECH2O-10HS (METER Group, Pullman, WA, USA)	[56,60,80,93,151]
	PH	E-201 (Shanghai REX Sensor Technology Co, Shanghai, China)	[96]
	Chemical elements (e.g.,: nitrate, nitrogen, etc.)	SEN0244 (DFROBOTS, Shangai, China)	[109]
Environment monitoring	Air temperature, air humidity	DHT11, DHT22 (AM2302, Aosong Electronics Co. Ltd., Guangzhou, China)	[104,114,154]
	Solar radiation	SQ-110 (Apogee Instruments, Inc., Logan, UT, USA)	[105]
	Rain	YF-S402 (Graylogix, Bangalore, Karnataka, India), YL-83 (Vaisala Corp., Helsinki, Finland) SE-WS700D (Lufft Inc., Berlin, Germany)	[27,124]
	Luminosity	BH1750 (Rohm Semiconductor, Kyoto, Japan), TSL2561 (Adafruit Industries, New York City, NY, USA)	[109,139]
	Atmospheric pressure	MPL3115A2 (NXP Semiconductors, Eindhoven, Netherlands)	[38]
	Wind speed and direction	WS-3000 (Ambient Weather, Chandler, AZ, USA), SEN08942 (SparkFun Electronics, Niwot, Colorado, USA)	[38,105]
	CO2 concentration	MG-811 (Zhengzhou Winsen Electronics Technology Co., Ltd., Zhengzhou, China), MQ135 (Waveshare Electronics, Shenzhen, China)	[96,104]
Other	Tracking	Mifare Ultralight NFC tag (NXP Semiconductors, Eindhoven, Netherlands), Blueberry RFID reader (Tertium Technology, Bangalore, Karnataka, India)	[168,169]
	Localization	UM220-III (Unicore Communication Inc., Beijing, China)	[75,181]

**Table 4:** Embedded system platforms and UAV devices in smart farming.

Application	Arduino	Raspberry	ESP	UAV
Disease prevention	[129,132]	[130]	[129,130]	[129]
Waste management		[145]		
Chemical control				[48,53]
Crop monitoring	[24,62,63,65,84,94,98,101,105,106,107,108,109,110,115,116,118,120,122,128]	[29,30,84,95,98,110,111,112,113,114,117,118]	[33,63,80,97,102,104,116,119,128]	[24,47,57,58,66,76,126,186]
Soil management	[159,162]	[30,156]		
Vehicles and Machinery control		[174]		
Irrigation control	[27,34,139,147,154,155]	[27,144]	[146,154,187]	

**Table 5:** Use of network protocols in smart farming for different farming scenarios.

Network Protocols		Arable Land	Generic	Greenhouse	Orchard
**Wired**	CAN		[145]	[100]	[56]
	Ethernet		[74,84]	[110,122]	
**Short range**	Bluetooth	[66]	[81,83,187]	[108,115,117]	[188]
	LoRa	[60,67,68,141]	[98,187]	[114,137]	[34]
	NFC				[188]
	RFID		[96,157,165,179]		
	ZigBee	[28,30,62,70,131,175]	[83,92,93,96,158,178,179,189]	[99,105,106,107,112,113,118]	[56,162,188]
**Middle Range**	(RF-ISM)	[24,69,70,144]	[147,179,189]	[103,166,180]	[124]
	Wi-Fi	[63,129,130,132,139,156]	[27,33,74,80,83,84,94,96,97,146,177]	[101,102,104,105,107,108,109,111,113,115,116,117,119,120]	
**Long range**	LoRaWAN	[68]	[84,90,98,135]	[114,137]	
	Cellular	[28,38,66,75,129,139,141,142,176]	[98,133,135,159]	[99,100,101,109,117]	[56,124,151]
	Sigfox				[34]

**Table 6:** IoT-enabling platforms and data processing technologies used in smart farming within the reviewed papers.

Platform	Artificial Intelligence	Big Data	Machine Learning	Computer Vision	Other/Not Identified
AgroCloud		[195]			
AT&T M2X Cloud					[163]
AWS	[156]	[142]	[142,156]		
Azure IoT Hub		[67]			[84]
Blynk					[139]
Cropinfra					[41]
Dropbox					[65,89]
ERMES					[77]
FIWARE					[90,92,141,143,150]
Freeboard					[98]
Google			[111]		
GroveStream					[98]
MACQU					[121]
Mobius	[95]				
NETPIE					[102]
Rural IoT		[30]	[30]		[30]
Self-developed	[181]	[79]	[144]		[64,85,114,143,174,196]
SmartFarmNET					[32]
Thinger.io		[33]			
ThingSpeak	[97]			[132]	[27,34,60,80,98,104,110,115,119,122]
Ubidots					[28,94,117,131]

**Table 7:** Technologies and application in smart farming.

Application	Artificial Intelligence	Big Data	Computer Vision	Machine Learning	Blockchain	Fuzzy Logic	Other Technologies
Disease Prevention	[134,137]		[129,132,133,134]	[133,137]			
Supply chain traceability		[164,165]			[167,171]		
Waste Management							[145]
Chemical control	[56]		[48]	[48,53]			
Crop monitoring	[25,95,97,125]	[30,33,67,79,113,118,125,194,197,200]	[58,62,76,124,125,126,200]	[25,30,47,63,73,82,83,86,87,93,111,115,118,126]	[30,91]	[64,73,107,120]	
Soil Management	[156,160,161]	[160,161]		[156,160]			
Vehicles and Machinery control							[41,172]
Irrigation control	[46]	[46,140,142,148,152]		[46,142,144]		[154,155]	[150]

## References

[1] United Nations, Department of Economic and Social Affairs, Population Division (2019). World Population Prospects 2019: Highlights.

[2] Satterthwaite D. (2009). The implications of population growth and urbanization for climate change. Environ. Urban..

[3] Walter A., Finger R., Huber R., Buchmann N. (2017). Opinion: Smart farming is key to developing sustainable agriculture. Proc. Natl. Acad. Sci. USA.

[4] Wolfert S., Ge L., Verdouw C., Bogaardt M.-J. (2017). Big Data in Smart Farming—A review. Agric. Syst..

[5] Pivoto D., Waquil P.D., Talamini E., Finocchio C.P.S., Corte V.F.D., de Mores G.V. (2018). Scientific development of smart farming technologies and their application in Brazil. Inf. Process. Agric..

[6] Madakam S., Ramaswamy R., Tripathi S. (2015). Internet of Things (IoT): A Literature Review. J. Comput. Commun..

[7] Leonard E.C. (2016). Precision Agriculture. Encyclopedia of Food Grains.

[8] Ibarra-Esquer J., González-Navarro F., Flores-Rios B., Burtseva L., Astorga-Vargas M. (2017). Tracking the Evolution of the Internet of Things Concept Across Different Application Domains. Sensors.

[9] BNDES Estudo “Internet das Coisas: Um plano de ação para o Brasil”. https://www.bndes.gov.br/wps/portal/site/home/conhecimento/pesquisaedados/estudos/estudo-internet-das-coisas-iot/estudo-internet-das-coisas-um-plano-de-acao-para-o-brasil.

[10] Zarco-Tejada P., Hubbard N., Loudjani P., Joint Research Centre (JRC) of the European Commission (2014). Precision Agriculture: An Opportunity for EU-Farmers—Potential Support with the CAP 2014–2020.

[11] Villa-Henriksen A., Edwards G.T.C., Pesonen L.A., Green O., Sørensen C.A.G. (2020). Internet of Things in arable farming: Implementation, applications, challenges and potential. Biosyst. Eng..

[12] Ray P.P. (2017). Internet of things for smart agriculture: Technologies, practices and future direction. J. Ambient Intell. Smart Environ..

[13] Talavera J.M., Tobón L.E., Gómez J.A., Culman M.A., Aranda J.M., Parra D.T., Quiroz L.A., Hoyos A., Garreta L.E. (2017). Review of IoT applications in agro-industrial and environmental fields. Comput. Electron. Agric..

[14] Tzounis A., Katsoulas N., Bartzanas T., Kittas C. (2017). Internet of Things in agriculture, recent advances and future challenges. Biosyst. Eng..

[15] Verdouw C.N., Wolfert J., Tekinerdogan B. (2016). Internet of Things in agriculture. CAB Rev. Perspect. Agric. Vet. Sci. Nutr. Nat. Resour..

[16] Bhagat M., Kumar D., Kumar D. Role of Internet of Things (IoT) in Smart Farming: A Brief Survey. Proceedings of the IEEE 2019 Devices for Integrated Circuit (DevIC).

[17] Stočes M., Vaněk J., Masner J., Pavlík J. (2016). Internet of Things (IoT) in Agriculture—Selected Aspects. Agris on-line Pap. Econ. Informat..

[18] Statista Forecasted Market Value of Precision Farming Worldwide in 2018 and 2023. https://www.statista.com/statistics/721921/forecasted-market-value-of-precision-farming-worldwide/.

[19] Kite-Powell J. Why Precision Agriculture Will Change How Food Is Produced. https://www.forbes.com/sites/jenniferhicks/2018/04/30/why-precision-agriculture-will-change-how-food-is-produced/#1aa438ec6c65.

[20] Zhao J.C., Zhang J.F., Feng Y., Guo J.X. The study and application of the IOT technology in agriculture. Proceedings of the IEEE 2010 3rd International Conference on Computer Science and Information Technology.

[21] Verdouw C., Sundmaeker H., Tekinerdogan B., Conzon D., Montanaro T. (2019). Architecture framework of IoT-based food and farm systems: A multiple case study. Comput. Electron. Agric..

[22] Chen J., Yang A. (2019). Intelligent Agriculture and Its Key Technologies Based on Internet of Things Architecture. IEEE Access.

[23] Telecommunication Standardization Sector of ITU (2012). Recommendation ITU-T Y.2060: Overview of the Internet of Things.

[24] Uddin M.A., Mansour A., Jeune D.L., Ayaz M., Aggoune E.-H.M. (2018). UAV-Assisted Dynamic Clustering of Wireless Sensor Networks for Crop Health Monitoring. Sensors.

[25] Liu N., Cao W., Zhu Y., Zhang J., Pang F., Ni J. (2016). Node Deployment with k-Connectivity in Sensor Networks for Crop Information Full Coverage Monitoring. Sensors.

[26] Thorat A., Kumari S., Valakunde N.D. An IoT based smart solution for leaf disease detection. Proceedings of the IEEE 2017 International Conference on Big Data, IoT and Data Science (BID).

[27] Rivas-Sánchez Y., Moreno-Pérez M., Roldán-Cañas J. (2019). Environment Control with Low-Cost Microcontrollers and Microprocessors: Application for Green Walls. Sustainability.

[28] Karim F., Karim F., Frihida A. (2017). Monitoring system using web of things in precision agriculture. Procedia Comput. Sci..

[29] Navulur S., Sastry A., Prasad M.G. (2017). Agricultural Management through Wireless Sensors and Internet of Things. Int. J. Electr. Comput. Eng..

[30] Alonso R.S., Sittón-Candanedo I., García Ó., Prieto J., Rodríguez-González S. (2020). An intelligent Edge-IoT platform for monitoring livestock and crops in a dairy farming scenario. Ad Hoc Netw..

[31] FIWARE The Open Source Platform for Our Smart Digital Future—FIWARE. https://www.fiware.org/.

[32] Jayaraman P., Yavari A., Georgakopoulos D., Morshed A., Zaslavsky A. (2016). Internet of Things Platform for Smart Farming: Experiences and Lessons Learnt. Sensors.

[33] Luis Bustamante A., Patricio M., Molina J. (2019). Thinger.io: An Open Source Platform for Deploying Data Fusion Applications in IoT Environments. Sensors.

[34] Fernández-Ahumada L.M., Ramírez-Faz J., Torres-Romero M., López-Luque R. (2019). Proposal for the Design of Monitoring and Operating Irrigation Networks Based on IoT, Cloud Computing and Free Hardware Technologies. Sensors.

[35] Mekki K., Bajic E., Chaxel F., Meyer F. (2019). A comparative study of LPWAN technologies for large-scale IoT deployment. ICT Express.

[36] Sanchez-Iborra R., Cano M.-D. (2016). State of the Art in LP-WAN Solutions for Industrial IoT Services. Sensors.

[37] Peterson L.L., Davie B.S. (2011). Computer Networks, Fifth Edition: A Systems Approach.

[38] Trilles S., González-Pérez A., Huerta J. (2018). A Comprehensive IoT Node Proposal Using Open Hardware. A Smart Farming Use Case to Monitor Vineyards. Electronics.

[39] Shelby Z., Hartke K., Bormann C. The Constrained Application Protocol (CoAP). https://www.rfc-editor.org/info/rfc7252.

[40] MQTT FAQ—Frequently Asked Questions: MQTT. http://mqtt.org/faq.

[41] Backman J., Linkolehto R., Koistinen M., Nikander J., Ronkainen A., Kaivosoja J., Suomi P., Pesonen L. (2019). Cropinfra research data collection platform for ISO 11783 compatible and retrofit farm equipment. Comput. Electron. Agric..

[42] Light R. MQTT Man Page. http://mosquitto.org/man/mqtt-7.html.

[43] Ray P.P. (2018). A survey on Internet of Things architectures. J. King Saud Univ. Comput. Inf. Sci..

[44] Chandra R., Prihastomo Y. (2012). Artificial Intelligence Definition: A Review. Master’s Thesis.

[45] Shi X., An X., Zhao Q., Liu H., Xia L., Sun X., Guo Y. (2019). State-of-the-Art Internet of Things in Protected Agriculture. Sensors.

[46] Adenugba F., Misra S., Maskeliūnas R., Damaševičius R., Kazanavičius E. (2019). Smart irrigation system for environmental sustainability in Africa: An Internet of Everything (IoE) approach. Math. Biosci. Eng..

[47] Li S., Yuan F., Ata-UI-Karim S.T., Zheng H., Cheng T., Liu X., Tian Y., Zhu Y., Cao W., Cao Q. (2019). Combining Color Indices and Textures of UAV-Based Digital Imagery for Rice LAI Estimation. Remote Sens..

[48] Lee S., Jeong Y., Son S., Lee B. (2019). A Self-Predictable Crop Yield Platform (SCYP) Based On Crop Diseases Using Deep Learning. Sustainability.

[49] Rowe E., Dawkins M.S., Gebhardt-Henrich S.G. (2019). A Systematic Review of Precision Livestock Farming in the Poultry Sector: Is Technology Focused on Improving Bird Welfare?. Animals.

[50] Bhakta I., Phadikar S., Majumder K. (2019). State-of-the-art technologies in precision agriculture: A systematic review. J. Sci. Food Agric..

[51] Parisoto G.J., Gil S.O., Schreinert I., De L., de Souza M., da Borba M.C., Gil S.O., Ramos J.E.S. (2018). Smart Farming e seu Estado da Arte: Uma Revisão Bibliométrica. Proceedings of the VI Simpósio da Ciência e do Agronegócio.

[52] Moher D., Liberati A., Tetzlaff J., Altman D.G. (2009). Preferred Reporting Items for Systematic Reviews and Meta-Analyses: The PRISMA Statement. J. Clin. Epidemiol..

[53] Zhai Z., Martínez Ortega J.-F., Lucas Martínez N., Rodríguez-Molina J. (2018). A Mission Planning Approach for Precision Farming Systems Based on Multi-Objective Optimization. Sensors.

[54] Cao Q., Miao Y., Shen J., Yuan F., Cheng S., Cui Z. (2018). Evaluating Two Crop Circle Active Canopy Sensors for In-Season Diagnosis of Winter Wheat Nitrogen Status. Agronomy.

[55] Zhang T., Zhou W., Meng F., Li Z. (2019). Efficiency Analysis and Improvement of an Intelligent Transportation System for the Application in Greenhouse. Electronics.

[56] Zhang X., Zhang J., Li L., Zhang Y., Yang G. (2017). Monitoring Citrus Soil Moisture and Nutrients Using an IoT Based System. Sensors.

[57] Han L., Yang G., Yang H., Xu B., Li Z., Yang X. (2018). Clustering Field-Based Maize Phenotyping of Plant-Height Growth and Canopy Spectral Dynamics Using a UAV Remote-Sensing Approach. Front. Plant Sci..

[58] Li S., Ding X., Kuang Q., Ata-UI-Karim S.T., Cheng T., Liu X., Tian Y., Zhu Y., Cao W., Cao Q. (2018). Potential of UAV-Based Active Sensing for Monitoring Rice Leaf Nitrogen Status. Front. Plant Sci..

[59] Ni J., Zhang J., Wu R., Pang F., Zhu Y. (2018). Development of an Apparatus for Crop-Growth Monitoring and Diagnosis. Sensors.

[60] Fisher D., Woodruff L., Anapalli S., Pinnamaneni S. (2018). Open-Source Wireless Cloud-Connected Agricultural Sensor Network. J. Sens. Actuator Netw..

[61] Muzafarov F., Eshmuradov A. (2019). Wireless sensor network based monitoring system for precision agriculture in Uzbekistan. TELKOMNIKA Telecommun. Comput. Electron. Control.

[62] Mateo-Aroca A., García-Mateos G., Ruiz-Canales A., Molina-García-Pardo J.M., Molina-Martínez J.M. (2019). Remote Image Capture System to Improve Aerial Supervision for Precision Irrigation in Agriculture. Water.

[63] Bhimanpallewar R., Rama Narasingarao M. (2018). A prototype model for continuous agriculture field monitoring and assessment. Int. J. Eng. Technol..

[64] Haseeb K., Din I.U., Almogren A., Islam N. (2020). An energy efficient and secure IoT-based WSN framework: An application to smart agriculture. Sensors.

[65] Thakur D., Kumar Y., Vijendra S. (2020). Smart Irrigation and Intrusions Detection in Agricultural Fields Using I.o.T. Procedia Comput. Sci..

[66] Idbella M., Iadaresta M., Gagliarde G., Mennella A., Mazzoleni S., Bonanomi G. (2020). Agrilogger: A new wireless sensor for monitoring agrometeorological data in areas lacking communication networks. Sensors.

[67] Symeonaki E., Arvanitis K., Piromalis D. (2020). A context-aware middleware cloud approach for integrating precision farming facilities into the IoT toward agriculture 4.0. Appl. Sci..

[68] Taskin D., Yazar S. (2020). A Long-range context-aware platform design for rural monitoring with IoT In precision agriculture. Int. J. Comput. Commun. Control.

[69] Liqiang Z., Shouyi Y., Leibo L., Zhen Z., Shaojun W. (2011). A Crop Monitoring System Based on Wireless Sensor Network. Procedia Environ. Sci..

[70] Zhang W., He Y., Liu F., Miao C., Sun S., Liu C., Jin J. (2012). Research on WSN Channel Fading Model and Experimental Analysis in Orchard Environment. IFIP Advances in Information and Communication Technology.

[71] Li Z., Wang J., Xu X., Zhao C., Jin X., Yang G., Feng H. (2015). Assimilation of Two Variables Derived from Hyperspectral Data into the DSSAT-CERES Model for Grain Yield and Quality Estimation. Remote Sens..

[72] Chen Y., Chanet J.-P., Hou K.-M., Shi H., de Sousa G. (2015). A Scalable Context-Aware Objective Function (SCAOF) of Routing Protocol for Agricultural Low-Power and Lossy Networks (RPAL). Sensors.

[73] Liu N., Cao W., Zhu Y., Zhang J., Pang F., Ni J. (2015). The Node Deployment of Intelligent Sensor Networks Based on the Spatial Difference of Farmland Soil. Sensors.

[74] Reynolds D., Ball J., Bauer A., Davey R., Griffiths S., Zhou J. (2019). CropSight: A scalable and open-source information management system for distributed plant phenotyping and IoT-based crop management. Gigascience.

[75] Zhang J., Hu J., Huang L., Zhang Z., Ma Y. (2016). A Portable Farmland Information Collection System with Multiple Sensors. Sensors.

[76] Ni J., Yao L., Zhang J., Cao W., Zhu Y., Tai X. (2017). Development of an Unmanned Aerial Vehicle-Borne Crop-Growth Monitoring System. Sensors.

[77] Granell C., Miralles I., Rodríguez-Pupo L., González-Pérez A., Casteleyn S., Busetto L., Pepe M., Boschetti M., Huerta J. (2017). Conceptual Architecture and Service-Oriented Implementation of a Regional Geoportal for Rice Monitoring. ISPRS Int. J. Geo Inf..

[78] Xing H., Xu X., Li Z., Chen Y., Feng H., Yang G., Chen Z. (2017). Global sensitivity analysis of the AquaCrop model for winter wheat under different water treatments based on the extended Fourier amplitude sensitivity test. J. Integr. Agric..

[79] Gunasekera K., Borrero A.N., Vasuian F., Bryceson K.P. (2018). Experiences in building an IoT infrastructure for agriculture education. Procedia Comput. Sci..

[80] Subashini M.M., Das S., Heble S., Raj U., Karthik R. (2018). Internet of Things based Wireless Plant Sensor for Smart Farming. Indones. J. Electr. Eng. Comput. Sci..

[81] Im H., Lee S., Naqi M., Lee C., Kim S. (2018). Flexible PI-Based Plant Drought Stress Sensor for Real-Time Monitoring System in Smart Farm. Electronics.

[82] Balducci F., Impedovo D., Pirlo G. (2018). Machine Learning Applications on Agricultural Datasets for Smart Farm Enhancement. Machines.

[83] Jeong Y., Son S., Lee S., Lee B. (2018). A Total Crop-Diagnosis Platform Based on Deep Learning Models in a Natural Nutrient Environment. Appl. Sci..

[84] Liu Y., Akram Hassan K., Karlsson M., Pang Z., Gong S. (2019). A Data-Centric Internet of Things Framework Based on Azure Cloud. IEEE Access.

[85] Cambra Baseca C., Sendra S., Lloret J., Tomas J. (2019). A Smart Decision System for Digital Farming. Agronomy.

[86] Murugesan R., Sudarsanam S.K., Malathi G., Vijayakumar V., Neelanarayanan V., Venugopal R., Rekha D., Saha S., Bajaj R., Miral A. (2019). Artificial Intelligence and Agriculture 5. 0. Int. J. Recent Technol. Eng..

[87] Jin X.B., Yu X.H., Wang X.Y., Bai Y.T., Su T.L., Kong J.L. (2020). Deep learning predictor for sustainable precision agriculture based on internet of things system. Sustainability.

[88] Lee K., Silva B.N., Han K. (2020). Deep learning entrusted to fog nodes (DLEFN) based smart agriculture. Appl. Sci..

[89] Murthy A.S.R., Sudheer Y., Mounika K., Rao K.S., Prasad P.D. (2016). Cloud Technology on Agriculture using Sensors. Indian J. Sci. Technol..

[90] Zyrianoff I., Heideker A., Silva D., Kleinschmidt J., Soininen J.P., Cinotti T.S., Kamienski C. (2020). Architecting and deploying IoT smart applications: A performance–oriented approach. Sensors.

[91] Awan S.H., Ahmed S., Nawaz A., Sulaiman S., Zaman K., Ali M.Y., Najam Z., Imran S. (2020). BlockChain with IoT, an Emergent Routing Scheme for Smart Agriculture. Int. J. Adv. Comput. Sci. Appl..

[92] Martínez R., Pastor J., Álvarez B., Iborra A. (2016). A Testbed to Evaluate the FIWARE-Based IoT Platform in the Domain of Precision Agriculture. Sensors.

[93] Geng L., Dong T. (2017). An agricultural monitoring system based on wireless sensor and depth learning algorithm. Int. J. Online Eng..

[94] Montoya E.A.Q., Colorado S.F.J., Muñoz W.Y.C., Golondrino G.E.C. (2017). Propuesta de una Arquitectura para Agricultura de Precisión Soportada en IoT. RISTI Rev. Ibérica Sist. e Tecnol. Informação.

[95] Patil S.M., Sakkaravarthi R. (2017). Internet of things based smart agriculture system using predictive analytics. Asian J. Pharm. Clin. Res..

[96] Guandong G., Yuchen J., Ke X. (2018). An IOT-based Multi-sensor Ecological Shared Farmland Management System. Int. J. Online Eng..

[97] Aliev K., Moazzam M., Narejo S., Pasero E., Pulatov A. (2018). Internet of Plants Application for Smart Agriculture. Int. J. Adv. Comput. Sci. Appl..

[98] Dupont C., Vecchio M., Pham C., Diop B., Dupont C., Koffi S. (2018). An Open IoT Platform to Promote Eco-Sustainable Innovation in Western Africa: Real Urban and Rural Testbeds. Wirel. Commun. Mob. Comput..

[99] Junxiang G., Haiqing D. (2011). Design of Greenhouse Surveillance System Based on Embedded Web Server Technology. Procedia Eng..

[100] Hong L.S., Sa Z.S., Yan J. (2016). Environment Factors Monitoring System Based on CAN bus. Int. J. Online Eng..

[101] Shasi Kiran U., Arya S., Rajasekaran M. (2018). Design and Implementation of Smart and Low Cost Multi-task Farming System Using Arduino. Int. J. Eng. Technol..

[102] Boonchieng E., Chieochan O., Saokaew A. (2018). Smart Farm: Applying the Use of NodeMCU, IOT, NETPIE and LINE API for a Lingzhi Mushroom Farm in Thailand. IEICE Trans. Commun..

[103] Cambra C., Sendra S., Lloret J., Lacuesta R. (2018). Smart System for Bicarbonate Control in Irrigation for Hydroponic Precision Farming. Sensors.

[104] Azimi Mahmud M.S., Buyamin S., Mokji M.M., Abidin M.S.Z. (2018). Internet of Things based Smart Environmental Monitoring for Mushroom Cultivation. Indones. J. Electr. Eng. Comput. Sci..

[105] Erazo-Rodas M., Sandoval-Moreno M., Muñoz-Romero S., Huerta M., Rivas-Lalaleo D., Naranjo C., Rojo-Álvarez J. (2018). Multiparametric Monitoring in Equatorian Tomato Greenhouses (I): Wireless Sensor Network Benchmarking. Sensors.

[106] Barbosa R.Z., Martins J.E.M.P. (2018). Design of a wireless sensor network for greenhouses temperature analysis. Irriga.

[107] Alpay Ö., Erdem E. (2018). The Control of Greenhouses Based on Fuzzy Logic Using Wireless Sensor Networks. Int. J. Comput. Intell. Syst..

[108] Munir M.S., Bajwa I.S., Naeem M.A., Ramzan B. (2018). Design and Implementation of an IoT System for Smart Energy Consumption and Smart Irrigation in Tunnel Farming. Energies.

[109] Syafarinda Y., Akhadin F., Fitri Z.E., Widiawan B., Rosdiana E. (2018). The Precision Agriculture Based on Wireless Sensor Network with MQTT Protocol. IOP Conf. Ser. Earth Environ. Sci..

[110] Radharamana K., Mouli C., Kumar U. (2019). Web Architecture for Monitoring Field using Representational State Transfer Methods. Int. J. Intell. Eng. Syst..

[111] Kumar V.S., Gogul I., Raj M.D., Pragadesh S.K., Sebastin J.S. (2016). Smart Autonomous Gardening Rover with Plant Recognition Using Neural Networks. Procedia Comput. Sci..

[112] Cama-Pinto D., Damas M., Holgado-Terriza J.A., Gómez-Mula F., Cama-Pinto A. (2019). Path Loss Determination Using Linear and Cubic Regression Inside a Classic Tomato Greenhouse. Int. J. Environ. Res. Public Health.

[113] Li X., Ma Z., Zheng J., Liu Y., Zhu L., Zhou N. (2020). An effective edge-assisted data collection approach for critical events in the SDWSN-based agricultural internet of things. Electronics.

[114] Codeluppi G., Cilfone A., Davoli L., Ferrari G. (2020). LoraFarM: A LoRaWAN-based smart farming modular IoT architecture. Sensors.

[115] Kim T.H., Solanki V.S., Baraiya H.J., Mitra A., Shah H., Roy S. (2020). A smart, sensible agriculture system using the exponential moving average model. Symmetry.

[116] Sarat Chandra G., Srinivas Ravi K. (2016). Effective Architecture for Greenhouse Controlling and Monitoring using Wi-Fi Peer to Peer Direct Protocol. Indian J. Sci. Technol..

[117] Ferrández-Pastor F., García-Chamizo J., Nieto-Hidalgo M., Mora-Pascual J., Mora-Martínez J. (2016). Developing Ubiquitous Sensor Network Platform Using Internet of Things: Application in Precision Agriculture. Sensors.

[118] Rodríguez S., Gualotuña T., Grilo C. (2017). A System for the Monitoring and Predicting of Data in Precision Agriculture in a Rose Greenhouse Based on Wireless Sensor Networks. Procedia Comput. Sci..

[119] Boonnam N., Pitakphongmetha J., Kajornkasirat S., Horanont T., Somkiadcharoen D., Prapakornpilai J. (2017). Optimal Plant Growth in Smart Farm Hydroponics System using the Integration of Wireless Sensor Networks into Internet of Things. Adv. Sci. Technol. Eng. Syst. J..

[120] Algarín C.R., Cabarcas J.C., Llanos A.P. (2017). Low-Cost Fuzzy Logic Control for Greenhouse Environments with Web Monitoring. Electronics.

[121] Li L., Li J., Wang H., Georgieva T., Ferentinos K.P., Arvanitis K.G., Sigrimis N.A. (2018). Sustainable energy management of solar greenhouses using open weather data on MACQU platform. Int. J. Agric. Biol. Eng..

[122] Laktionov I.S., Vovna O.V., Bashkov Y.O., Zori A.A., Lebediev V.A. (2019). Improved Computer-oriented Method for Processing of Measurement Information on Greenhouse Microclimate. Int. J. Bioautomation.

[123] Villalba G., Plaza F., Zhong X., Davis T., Navarro M., Li Y., Slater T., Liang Y., Liang X. (2017). A Networked Sensor System for the Analysis of Plot-Scale Hydrology. Sensors.

[124] Kameoka S., Isoda S., Hashimoto A., Ito R., Miyamoto S., Wada G., Watanabe N., Yamakami T., Suzuki K., Kameoka T. (2017). A Wireless Sensor Network for Growth Environment Measurement and Multi-Band Optical Sensing to Diagnose Tree Vigor. Sensors.

[125] Xia J., Huang B., Yang Y.W., Cao H.X., Zhang W., Xu L., Wan Q., Ke Y., Zhang W., Ge D. (2018). Hyperspectral Identification and Classification of Oilseed Rape Waterlogging Stress Levels Using Parallel Computing. IEEE Access.

[126] Xue J., Fan Y., Su B., Fuentes S. (2019). Assessment of canopy vigor information from kiwifruit plants based on a digital surface model from unmanned aerial vehicle imagery. Int. J. Agric. Biol. Eng..

[127] Lin H., Cai K., Chen H., Zeng Z. (2015). The Construction of a Precise Agricultural Information System Based on Internet of Things. Int. J. Online Eng..

[128] Shashi Rekha N., Kousar Nikhath A., Nagini S., Sagar Y., Sukheja D. (2019). Sustainable and Portable Low Cost IOT Based Terrace Model to Grow True Organic Greens. Int. J. Eng. Adv. Technol..

[129] Kumar S., Mishra S., Khanna P. (2017). Pragya Precision Sugarcane Monitoring Using SVM Classifier. Procedia Comput. Sci..

[130] Pérez-Expósito J., Fernández-Caramés T., Fraga-Lamas P., Castedo L. (2017). VineSens: An Eco-Smart Decision-Support Viticulture System. Sensors.

[131] Foughali K., Fathallah K., Frihida A. (2018). Using Cloud IOT for disease prevention in precision agriculture. Procedia Comput. Sci..

[132] Gayathri Devi T., Srinivasan A., Sudha S., Narasimhan D. (2019). Web enabled paddy disease detection using Compressed Sensing. Math. Biosci. Eng..

[133] Kalamatianos R., Karydis I., Doukakis D., Avlonitis M. (2018). DIRT: The Dacus Image Recognition Toolkit. J. Imaging.

[134] Lammie C., Olsen A., Carrick T., Rahimi Azghadi M. (2019). Low-Power and High-Speed Deep FPGA Inference Engines for Weed Classification at the Edge. IEEE Access.

[135] Potamitis I., Rigakis I., Tatlas N.-A., Potirakis S. (2019). In-Vivo Vibroacoustic Surveillance of Trees in the Context of the IoT. Sensors.

[136] Fathallah K., Abid M.A., Hadj-Alouane N. (2020). Ben Enhancing Energy Saving in Smart Farming through Aggregation and Partition Aware IOT Routing Protocol. Sensors.

[137] Kim S., Lee M., Shin C. (2018). IoT-Based Strawberry Disease Prediction System for Smart Farming. Sensors.

[138] Potamitis I., Eliopoulos P., Rigakis I. (2017). Automated Remote Insect Surveillance at a Global Scale and the Internet of Things. Robotics.

[139] Mohanraj I., Ashokumar K., Naren J. (2016). Field Monitoring and Automation Using IOT in Agriculture Domain. Procedia Comput. Sci..

[140] González-Briones A., Castellanos-Garzón J.A., Mezquita Martín Y., Prieto J., Corchado J.M. (2018). A Framework for Knowledge Discovery from Wireless Sensor Networks in Rural Environments: A Crop Irrigation Systems Case Study. Wirel. Commun. Mob. Comput..

[141] Kamienski C., Soininen J.-P., Taumberger M., Dantas R., Toscano A., Salmon Cinotti T., Filev Maia R., Torre Neto A. (2019). Smart Water Management Platform: IoT-Based Precision Irrigation for Agriculture. Sensors.

[142] Revathi N., Sengottuvelan P. (2019). Real-Time Irrigation Scheduling Through IoT in Paddy Fields. Int. J. Innov. Technol. Explor. Eng..

[143] López-Morales J.A., Martínez J.A., Skarmeta A.F. (2020). Digital transformation of agriculture through the use of an interoperable platform. Sensors.

[144] Campos N.G.S., Rocha A.R., Gondim R., da Silva T.L.C., Gomes D.G. (2020). Smart & green: An internet-of-things framework for smart irrigation. Sensors.

[145] Nikoloudakis Y., Panagiotakis S., Manios T., Markakis E., Pallis E. (2018). Composting as a Service: A Real-World IoT Implementation. Futur. Internet.

[146] Kamelia L., Ramdhani M.A., Faroqi A., Rifadiapriyana V. (2018). Implementation of Automation System for Humidity Monitoring and Irrigation System. IOP Conf. Ser. Mater. Sci. Eng..

[147] Sheikh S.S., Javed A., Anas M., Ahmed F. (2018). Solar Based Smart Irrigation System Using PID Controller. IOP Conf. Ser. Mater. Sci. Eng..

[148] Jarolímek J., Pavlík J., Kholova J., Ronanki S. (2019). Data Pre-processing for Agricultural Simulations. Agris on-line Pap. Econ. Inform..

[149] Abd Rahman M.K.I., Zainal Abidin M.S., Buyamin S., Azimi Mahmud M.S. (2018). Enhanced Fertigation Control System towards Higher Water Saving Irrigation. Indones. J. Electr. Eng. Comput. Sci..

[150] Muñoz M., Gil J.D., Roca L., Rodríguez F., Berenguel M. (2020). An iot architecture for water resource management in agroindustrial environments: A case study in almería (Spain). Sensors.

[151] Domínguez-Niño J.M., Oliver-Manera J., Girona J., Casadesús J. (2020). Differential irrigation scheduling by an automated algorithm of water balance tuned by capacitance-type soil moisture sensors. Agric. Water Manag..

[152] Ruan F., Gu R., Huang T., Xue S. (2019). A big data placement method using NSGA-III in meteorological cloud platform. Eurasip J. Wirel. Commun. Netw..

[153] Mao Y., Qi H., Ping P., Li X. (2017). Contamination Event Detection with Multivariate Time-Series Data in Agricultural Water Monitoring. Sensors.

[154] Karimah S.A., Rakhmatsyah A., Suwastika N.A. (2019). Smart pot implementation using fuzzy logic. J. Phys. Conf. Ser..

[155] Mungai Bryan N., Fei Thang K., Vinesh T. (2019). An Urban Based Smart IOT Farming System. IOP Conf. Ser. Earth Environ. Sci..

[156] Vincent D.R., Deepa N., Elavarasan D., Srinivasan K., Chauhdary S.H., Iwendi C. (2019). Sensors Driven AI-Based Agriculture Recommendation Model for Assessing Land Suitability. Sensors.

[157] Aroca R.V., Hernandes A.C., Magalhães D.V., Becker M., Vaz C.M.P., Calbo A.G. (2018). Calibration of Passive UHF RFID Tags Using Neural Networks to Measure Soil Moisture. J. Sensors.

[158] Burton L., Dave N., Fernandez R.E., Jayachandran K., Bhansali S. (2018). Smart Gardening IoT Soil Sheets for Real-Time Nutrient Analysis. J. Electrochem. Soc..

[159] Cruz F.R.G., Ballado A.H., Alcala A.K.A., Legaspi A.K.S., Lozada E.L., Portugal V.L.P. Wireless soil moisture detection with time drift compensation. Proceedings of the AIP Conference Proceedings.

[160] Jain N. (2019). WSN-AI based Cloud Computing Architectures for Energy Efficient Climate Smart Agriculture with Big Data analysis. Int. J. Adv. Trends Comput. Sci. Eng..

[161] Figueroa M., Pope C. (2017). Root System Water Consumption Pattern Identification on Time Series Data. Sensors.

[162] Zervopoulos A., Tsipis A., Alvanou A.G., Bezas K., Papamichail A., Vergis S., Stylidou A., Tsoumanis G., Komianos V., Koufoudakis G. (2020). Wireless sensor network synchronization for precision agriculture applications. Agriculture.

[163] Divya Vani P., Raghavendra Rao K. (2016). Measurement and Monitoring of Soil Moisture using Cloud IoT and Android System. Indian J. Sci. Technol..

[164] Srinivasan S.P., Anitha J., Vijayakumar R. (2017). Integration of internet of things to reduce various losses of jatropha seed supply chain. IOP Conf. Ser. Mater. Sci. Eng..

[165] Jiang W. (2019). An Intelligent Supply Chain Information Collaboration Model Based on Internet of Things and Big Data. IEEE Access.

[166] Tervonen J. (2018). Experiment of the quality control of vegetable storage based on the Internet-of-Things. Procedia Comput. Sci..

[167] Borrero J.D. (2019). Sistema de trazabilidad de la cadena de suministro agroalimentario para cooperativas de frutas y hortalizas basado en la tecnología Blockchain. CIRIEC-España Rev. Econ. Pública Soc. y Coop..

[168] Mainetti L., Mele F., Patrono L., Simone F., Stefanizzi M.L., Vergallo R. (2013). An RFID-Based Tracing and Tracking System for the Fresh Vegetables Supply Chain. Int. J. Antennas Propag..

[169] Ko D., Kwak Y., Song S. (2014). Real Time Traceability and Monitoring System for Agricultural Products Based on Wireless Sensor Network. Int. J. Distrib. Sens. Netw..

[170] Fan H. (2019). Theoretical Basis and System Establishment of China Food Safety Intelligent Supervision in the Perspective of Internet of Things. IEEE Access.

[171] Liao Y., Xu K. (2019). Traceability System of Agricultural Product Based on Block-chain and Application in Tea Quality Safety Management. J. Phys. Conf. Ser..

[172] Huang T., Yan S., Yang F., Pan T., Liu J. (2016). Building SDN-Based Agricultural Vehicular Sensor Networks Based on Extended Open vSwitch. Sensors.

[173] Tsolakis N., Bechtsis D., Bochtis D. (2019). AgROS: A Robot Operating System Based Emulation Tool for Agricultural Robotics. Agronomy.

[174] Touseau L., Sommer N. (2019). Contribution of the Web of Things and of the Opportunistic Computing to the Smart Agriculture: A Practical Experiment. Futur. Internet.

[175] Gao Z., Li W., Zhu Y., Tian Y., Pang F., Cao W., Ni J. (2018). Wireless Channel Propagation Characteristics and Modeling Research in Rice Field Sensor Networks. Sensors.

[176] Jin X.B., Yang N.X., Wang X.Y., Bai Y.T., Su T.L., Kong J.L. (2020). Hybrid deep learning predictor for smart agriculture sensing based on empirical mode decomposition and gated recurrent unit group model. Sensors.

[177] Dhall R., Agrawal H. (2018). An Improved Energy Efficient Duty Cycling Algorithm for IoT based Precision Agriculture. Procedia Comput. Sci..

[178] Jawad H., Nordin R., Gharghan S., Jawad A., Ismail M., Abu-AlShaeer M. (2018). Power Reduction with Sleep/Wake on Redundant Data (SWORD) in a Wireless Sensor Network for Energy-Efficient Precision Agriculture. Sensors.

[179] Li C., Niu B. (2020). Design of smart agriculture based on big data and Internet of things. Int. J. Distrib. Sens. Netw..

[180] Sabri N., Mohammed S.S., Fouad S., Syed A.A., Al-Dhief F.T., Raheemah A. (2018). Investigation of Empirical Wave Propagation Models in Precision Agriculture. MATEC Web Conf..

[181] González-González M.G., Gómez-Sanchis J., Blasco J., Soria-Olivas E., Chueca P. (2020). CitrusYield: A dashboard for mapping yield and fruit quality of citrus in precision agriculture. Agronomy.

[182] Watanabe M., Nakamura A., Kunii A., Kusano K., Futagawa M. (2015). Fabrication of Scalable Indoor Light Energy Harvester and Study for Agricultural IoT Applications. J. Phys. Conf. Ser..

[183] García-Magariño I., Lacuesta R., Lloret J. (2018). ABS-SmartComAgri: An Agent-Based Simulator of Smart Communication Protocols in Wireless Sensor Networks for Debugging in Precision Agriculture. Sensors.

[184] Arduino Getting Started with Arduino Products. https://www.arduino.cc/en/Guide/HomePage.

[185] Raspberry Foundation Raspberry Pi: Products. https://www.raspberrypi.org/products/.

[186] Castellanos G., Deruyck M., Martens L., Joseph W. (2020). System Assessment of WUSN Using NB-IoT UAV-Aided Networks in Potato Crops. IEEE Access.

[187] Ali A.H., Chisab R.F., Mnati M.J. (2019). A smart monitoring and controlling for agricultural pumps using LoRa IOT technology. Indones. J. Electr. Eng. Comput. Sci..

[188] Wan X.-F., Yi Y., Zhang T., Zhang J.-W., Sardar M.S. (2018). Design of Distributed Agricultural Service Node with Smartphone In-field Access Supporting for Smart Farming in Beijing-Tianjin-Hebei Region. Sens. Mater..

[189] Klaina H., Vazquez Alejos A., Aghzout O., Falcone F. (2018). Narrowband Characterization of Near-Ground Radio Channel for Wireless Sensors Networks at 5G-IoT Bands. Sensors.

[190] Gungor V.C., Sahin D., Kocak T., Ergut S., Buccella C., Cecati C., Hancke G.P. (2011). Smart Grid Technologies: Communication Technologies and Standards. IEEE Trans. Ind. Inform..

[191] Lea P. (2018). Long-Range Communication Systems and Protocols (WAN). Internet of Things for Architects: Learn to Design, Implement and secure your IoT infrastructure.

[192] Zhang Y., Li W.W. (2019). Energy Consumption Analysis of a Duty Cycle Wireless Sensor Network Model. IEEE Access.

[193] Xu D., Wu S., Zhang B., Qin X. (2013). Power Balance AODV Algorithm of WSN in Agriculture Monitoring. TELKOMNIKA Telecommun. Comput. Electron. Control.

[194] De Mauro A., Greco M., Grimaldi M. (2016). A formal definition of Big Data based on its essential features. Libr. Rev..

[195] Kodati S., Jeeva S. (2019). Smart Agricultural using Internet of Things, Cloud and Big Data. Int. J. Innov. Technol. Explor. Eng..

[196] Zhao L., He L., Jin X., Yu W. (2013). Design of Wireless Sensor Network Middleware for Agricultural Applications. IFIP Advances in Information and Communication Technology.

[197] Adam Ibrahim Fakherldin M., Adam K., Akma Abu Bakar N., Abdul Majid M. (2019). Weather Data Analysis Using Hadoop: Applications and Challenges. IOP Conf. Ser. Mater. Sci. Eng..

[198] NASA POWER, Data Sets from NASA Research with Solar and Meteorological Information. https://data.nasa.gov/Earth-Science/Prediction-Of-Worldwide-Energy-Resources-POWER-/wn3p-qsan.

[199] Steele J. Understanding Virtual Sensors: From Sensor Fusion To Context-Aware Applications.. http://electronicdesign.com/ios/understanding-virtual-sensors-sensor-fusion-context-aware-applications.

[200] Zhang L., Gui G., Khattak A.M., Wang M., Gao W., Jia J. (2019). Multi-Task Cascaded Convolutional Networks Based Intelligent Fruit Detection for Designing Automated Robot. IEEE Access.

[201] Chien Y.-R., Chen Y.-X. (2018). An RFID-Based Smart Nest Box: An Experimental Study of Laying Performance and Behavior of Individual Hens. Sensors.

